# Family cohesion, shame-proneness, expressive suppression, and adolescent mental health—A path model approach

**DOI:** 10.3389/fpsyg.2022.921250

**Published:** 2022-08-03

**Authors:** Rahel L. van Eickels, Achilleas Tsarpalis-Fragkoulidis, Martina Zemp

**Affiliations:** Department of Clinical and Health Psychology, University of Vienna, Vienna, Austria

**Keywords:** family system, shame, emotion regulation, psychopathology, adolescence, path analysis

## Abstract

**Objective:**

The family remains one of the most important relationship systems into early adulthood and provides an important foundation for lifelong mental health. Dysfunctional family cohesion can promote adjustment problems in adolescents and might also affect adolescents’ self-concept and strategies for coping with emotional distress. To test these relationships and the underlying mechanisms, we proposed a dual mediation model describing the associations between family cohesion and internalizing and externalizing problems, mediated by shame-proneness and expressive suppression.

**Methods:**

A sample of 526 German-speaking adolescents aged 14 to 18 years from Austria, Germany, and Switzerland participated in an online self-report survey encompassing questionnaires on family cohesion, shame-proneness, expressive suppression, and psychological problems. We tested a path model to examine the indirect pathways of the associations between family cohesion and internalizing and externalizing problems via shame-proneness and expressive suppression, while controlling for age, gender, and guilt-proneness.

**Results:**

We found a significant dual mediation of the associations between family cohesion and internalizing and externalizing problems by shame-proneness and expressive suppression. The indirect pathways were all significant, except for the indirect pathway from family cohesion to externalizing problems via shame-proneness.

**Discussion:**

Our results provide a model for the mechanisms by which disrupted family cohesion can be related to psychological problems in adolescents. Expressive suppression emerged as crucial when considering the consequences of shame-proneness in adolescents, as it was only indirectly related to externalizing problems via expressive suppression.

## Introduction

**Figure 1:**
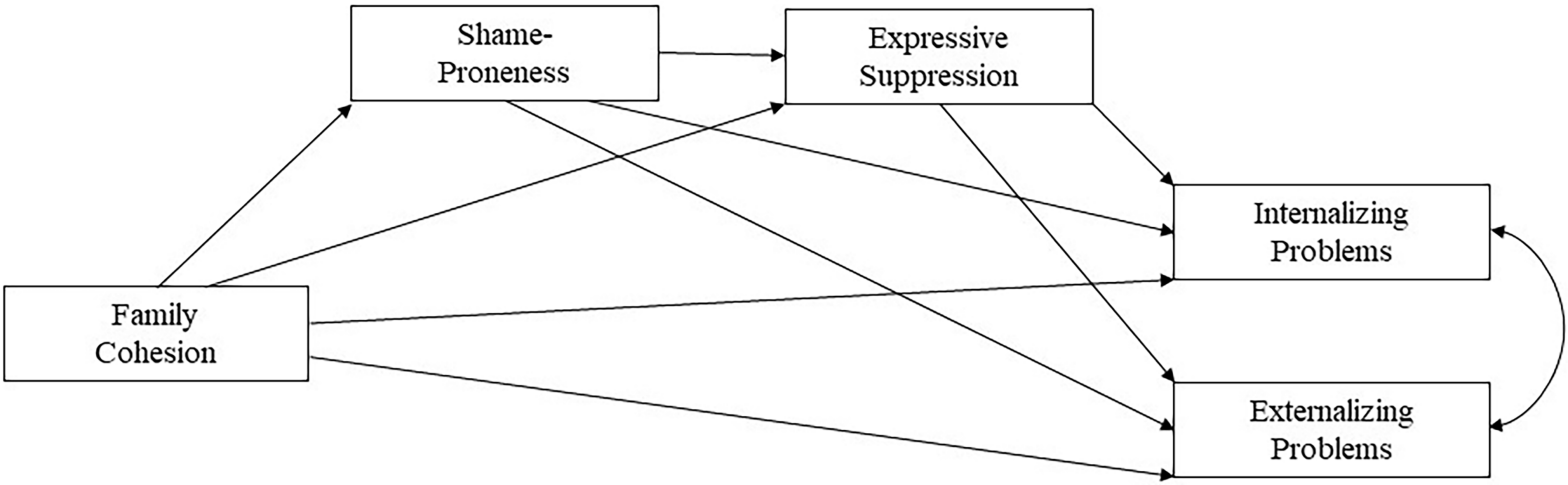
Theoretical model. Control variables are not displayed.

Adolescence is an important yet challenging time for self-development. As cognitive abilities increase, the self becomes more differentiated and abstract. This development process can also be accompanied by uncertainty, which can lead to fluctuations in self-evaluation (e.g., shame), impede emotion regulation, and may ultimately give rise to mental health problems (Harter, 2006; Szentágotai-Tătar and Miu, 2016; Parise et al., 2019). Emotional and behavioral problems in adolescence pose a serious health risk worldwide. In the German-speaking countries, the prevalence of mental disorders ranges between 17.5 and 35.8% (Eschmann et al., 2007; Fuchs and Karwautz, 2017; Steffen et al., 2018). Despite the increasing importance of peer relationships during adolescence, the family remains one of the most important relationship systems in this period (Laursen and Collins, 2009). Family functioning is crucial for youth well-being (Chen et al., 2017), and dysfunction in the family environment can promote psychopathology (Repetti et al., 2002; Rabinowitz et al., 2016; Murphy et al., 2017; Simpson et al., 2018). In the present study, we therefore investigate the mediating role of shame-proneness and expressive suppression in the association between family cohesion and mental health problems in adolescents, using a dual mediation model (see Figure 1).

Family functioning manifests itself in different aspects of family life, such as family cohesion, flexibility, communication, roles, and conflict (Hughes and Gullone, 2008). Family systems theory (FST) recognizes the family as a system of relationships that might be better understood in its wholeness rather than its parts (Cox and Paley, 2003). Various family systems theories emphasize emotional boundaries and connectedness between family members as an important feature of family functioning (Olson, 1989; Johnson and Waldo, 1998). The Circumplex Model of Family Systems (Olson, 2000), an integrative model of several systemic family theories used to identify families at risk of mental health problems, focuses on the three central dimensions of cohesion, flexibility and communication. In this model, the concept of family cohesion refers to emotional boundaries and connectedness. The model assumes a curvilinear structure of adaptivity, with the middle levels being the most adaptive and the extremes being dysfunctional. Thus, the emotional connections in the family can either be balanced (balanced cohesion in the middle), too permeable and overinvolved (enmeshed as one extreme), or too separated and uninvolved (disengaged as the other extreme; Olson, 2000).

Dysfunctional levels of family cohesion are associated with mental health problems. On the one hand, family enmeshment, marked by low autonomy of family members, high control, and permeable boundaries, is associated with adolescent internalizing and externalizing disorders (Barber and Buehler, 1996), eating disorders (Cerniglia et al., 2017), anxiety, and depression (Stark et al., 1990; Rowsell et al., 2016). On the other hand, disengaged families are characterized by strict boundaries, but the relationships are marked by low emotional warmth and involvement of the family members (Olson, 2000). Extreme forms of disengagement may even fall into the category of child maltreatment in the form of emotional neglect (Wark et al., 2003). Emotional neglect and (less severe) family disengagement are associated with anxiety and mood disorders (Young et al., 2011), conduct disorders (Coll et al., 2008; Young et al., 2011), eating disorders (Tafà et al., 2017), and suicidal behavior (Miller et al., 1992) in adolescents.

Previous research has already described several potential self-and emotion-related mediators that may explain the link between family dysfunction and adolescents’ mental health problems. These include dispositional shame (Murray et al., 2000; O’Leary et al., 2019) and emotion regulation (Morris et al., 2017), which we examine in more detail in the present study.

Shame is an aversive self-evaluation, as it represents a global devaluation of the self (“I am a bad person”). The concept of shame must not be confused with guilt, which evaluates a faulty action (“I did a bad thing”; Lewis, 1971). According to this conceptualization, these two emotions can be distinguished by the degree of stability and globality of attributions following a negative event. Individuals who tend to view their failures as products of an internal, stable, and global trait are more likely to experience shame, whereas guilt tends to be associated with internal, specific, and unstable attributions (Tracy and Robins, 2004; Carpenter et al., 2016). Given these underlying processes, it is not surprising that shame, which involves an inherent assault on core features of the self, leads to more avoidance behavior, such as withdrawal, denial, or escape. Guilt, on the other hand, involves more behavior-specific attributions and may lead to more reparative actions, such as apologies and attempts to undo the harm caused (Dearing et al., 2005; Tangney et al., 2007). The extent to which a person feels shame and guilt further ranges from normal-occasional to frequent-dispositional. The latter is referred to in the literature as shame-or guilt-proneness, i.e., the predisposition to experience shame or guilt across different situations and in various contexts (Lewis, 1971).

It is worth noting that shame and guilt are not always strictly adaptive or maladaptive, as it is often presented in the literature (Manion, 2002; Leach, 2017). Indeed, shame has been found to be associated with an increased drive for self-improvement and cooperative behavior when the situational context allows for it (de Hooge et al., 2008, 2010). Furthermore, a meta-analysis has shown that shame is associated with constructive approach orientations, when the failure that triggered the emotion is viewed as remediable (Leach and Cidam, 2015). Moreover, shame associated with moral failures can also lead to prosocial responses, when the feeling of shame relates to a specific self-defect rather than the whole self (Gausel et al., 2016). Analogously, guilt can also be understood as a maladaptive response, when it is caused by a distorted perception of responsibility in ambivalent situations or situations over which a person has no control (Tangney et al., 2007; Cândea and Szentagotai-Tătar, 2018). In sum, shame and guilt can be both adaptive and maladaptive responses to experiences of failure, depending on the context and accompanying cognitions.

That said, shame-proneness, defined as a general tendency to feel shame in various situations and assign the cause of one’s failure in a stable and global manner—as it is conceptualized and assessed in the present study— has consistently been shown to be maladaptive from a psychological viewpoint. Specifically, shame-proneness has been associated with mental health problems from childhood through adulthood, including, but not limited to, depression, anxiety, borderline personality disorder, aggression, and eating disorders (Tangney et al., 1992; Stuewig and McCloskey, 2005; Kim et al., 2011; Muris and Meesters, 2014; Velotti et al., 2014; Cesare et al., 2016; Cândea and Szentagotai-Tătar, 2018; Buchman-Wildbaum et al., 2021). This is, however, not true for guilt-proneness, which has either small or no associations with psychopathology, especially when accounting for shame (Tangney et al., 1992; Cândea and Szentagotai-Tătar, 2018).

Shame is difficult to regulate and has been linked to “maladaptive” emotion regulation strategies such as expressive suppression (Elison et al., 2006b; Schoenleber and Berenbaum, 2012; Szentágotai-Tătar and Miu, 2016). Expressive suppression refers to individuals’ attempts to conceal their emotions after an emotional response has been triggered (Gross and John, 2003). Although necessary in some contexts (Gross and Cassidy, 2019), expressive suppression is often deemed counterproductive, as it can intensify, rather than reduce, the subjective experience of negative emotions while dampening the experience of positive affect (Campbell-Sills et al., 2014; Dryman and Heimberg, 2018). These difficulties in emotion regulation may then carry over to other life domains and can lead to a wide array of emotional, behavioral, and social problems (Morris et al., 2007; Riediger and Klipker, 2014). Indeed, expressive suppression has been shown to be significantly associated with internalizing and externalizing problems (e.g., depressive and anxiety symptoms, self-injury, eating disorders, relational aggression; Gross and Cassidy, 2019).

Both shame-proneness and expressive suppression have been found to be predictors of psychopathology in adolescents (Rollins and Crandall, 2021), and since shame is particularly difficult to regulate, a mediation effect is also plausible. Difficulties in regulating shame are important contributors to personality pathology (Schoenleber and Berenbaum, 2012). In a similar vein, general difficulties in emotion regulation were found to mediate the effects of shame-proneness on eating disorder symptoms in women (Gupta et al., 2008), and expressive suppression mediated the influence of shame on psychopathological distress and hostility in female adolescents (Velotti et al., 2017).

Adolescence is a salient developmental stage for both shame and emotion dysregulation (Szentágotai-Tătar and Miu, 2016). Feelings of shame might emerge more frequently in adolescents due to the increased importance of social (e.g., peer) feedback (Gilbert and Irons, 2008). Research on the use of expressive suppression during adolescence has yielded mixed findings. However, it might be suggested that the need to regulate the generally more frequent and intense emotions within this age period paves the way for this regulation strategy (Spear, 2009; Gross and Cassidy, 2019). Therefore, a thorough investigation of such mechanisms among adolescents is necessary. The family might provide a developmental framework for the socialization of both shame and emotion regulation.

Although shame is an inherently intersubjective phenomenon, its social precursors are not entirely understood. Psychoanalysis views shame as a function of the superego, which is itself formed through parental disapproval and the striving for parental love (Rothstein, 1994). Indeed, family relationships, parenting, and a child’s attachment to the primary caregivers seem to play an important role in the development of self-conscious emotions and further dispositions to feel guilt and shame (Loader, 1998; Gross and Hansen, 2000; Muris et al., 2014). Specifically, negative parenting, such as rejecting, neglectful, controlling, shaming, or punitive behavior, is associated with children’s shame-proneness (Loader, 1998; Stuewig and McCloskey, 2005; Smiley et al., 2020). Other dysfunctional aspects of family life, such as boundary disruptions and inflexible family rules, may likewise elicit shame in children (Talmon and Ginzburg, 2017; Crane et al., 2020). However, the few available findings on the associations between family cohesion and shame-proneness are mixed. While one study found low cohesion to be related to higher shame in adults (Pulakos, 1996), another reported no effect of general family cohesion on shame with the exception of sibling-closeness, but did find an effect on guilt (Walter and Burnaford, 2006). Emotional neglect during childhood, by contrast, has been linked to child and adult shame-proneness (Bennett et al., 2010; Wojcik et al., 2019). A lack of family care might foster negative self-representations, which in turn might lead to shame (Kealy et al., 2020). Conversely, parental overprotection and psychological control have also been associated with shame-proneness in children, as these parenting styles might lead to children feeling weak and not in control of a situation, or might even expose them to direct devaluation (i.e., shaming and love withdrawal; Mills, 2005). Furthermore, a study examining undergraduate students’ feelings of guilt and shame and their retrospective reports of their parents’ disciplinary practices revealed that maternal affective control was associated with shame-proneness, while paternal affective control was not (Abell and Gecas, 1997).

Similarly, research has shown that family factors influence the development of emotion regulation during childhood (Morris et al., 2007; Thompson, 2014; Gross and Cassidy, 2019). Parents’ low responsiveness to children’s emotional distress, as well as their tendency to suppress their own emotions, can shape children’s perception of negative emotional experiences, leading them to believe that negative emotions are to be avoided rather than expressed (Eisenberg, 1996; Trosper et al., 2009; Bariola et al., 2012). Children whose parents do not provide them with appropriate autonomy in emotional situations lack the practice that is necessary for them to master the ability to regulate their emotions (Southam-Gerow and Kendall, 2002). Moreover, parents who tend to display controlling or overprotective behaviors when their children are in emotional distress discourage their children from experimenting with various emotion regulation strategies and developing appropriate strategies to regulate negative emotions (Fox and Calkins, 2003). Conversely, parents who accept and respond appropriately to children’s expressions of positive and negative emotions create a family environment that encourages children to freely communicate their feelings and thereby promotes the development of adaptive emotion regulation strategies (Gross and Cassidy, 2019). On a broader level, the family climate shows a direct association with child emotion regulation (Fosco and Grych, 2013). Specifically, family cohesion was shown to be related to adaptive anger regulation in adolescents (Houltberg et al., 2012), but enmeshment was linked to emotional dysregulation (Kivisto et al., 2015). A cold emotional family climate was also related to deficits in children’s use of adaptive emotion regulation strategies (Morris et al., 2007), and lower parental care was linked to children’s greater use of suppression (Jaffe et al., 2010). In a disengaged family environment, suppressing or avoiding intense emotions can be an adaptive way of dealing with emotional arousal, given that open expressions of emotion might be ignored or even punished (Repetti et al., 2002; Southam-Gerow and Kendall, 2002).

To the best of our knowledge, no study has yet investigated the outlined relationships between family cohesion, shame-proneness, expressive suppression, and internalizing and externalizing problems among adolescents within one cohesive model. Moreover, as many previous studies relied on adults’ retrospective perspectives, it is necessary to examine these associations in an adolescent sample.

Thus, the goal of this study was to examine the associations between family cohesion and internalizing and externalizing problems in adolescents, as well as the potential mediating roles of shame-proneness and expressive suppression. According to Olson (2000), dysfunctional family cohesion comprises high levels of either enmeshment or disengagement (for the purpose of this study, we summarize these into one score from dysfunctional to functional family cohesion; see below). Thus, we assumed negative links between (functional) family cohesion and adolescents’ shame-proneness, expressive suppression, internalizing problems and externalizing problems, and further hypothesized a dual mediation through shame-proneness and expressive suppression in these pathways (see Figure 1). Age, gender, and guilt-proneness were added to our model as control variables.

## Materials and methods

### Participants

The sample was drawn from the general German-speaking adolescent (14–18 years) population in Austria, Germany, and Switzerland. Participants were recruited using various methods and channels, including advertisements in newsletters and via social networks (e.g., Facebook). Out of 1,027 individuals who started the survey, 935 met the inclusion criterion of age (14–18 years). Of these, only 571 individuals completed the entire survey. We then excluded 24 individuals (4.2%) who completed the questionnaire in less than ten minutes, which, according to our test, was the minimum for a German-speaking participant to complete the questionnaire seriously. We further excluded ten individuals with a non-traditional family environment (i.e., living alone, with a partner, in foster care; 1.8%), and another 11 individuals (1.9%) due to missing data in central study variables. Thus, the final sample consisted of 526 adolescents (76.4% female) with a mean age of 15.74 years (*SD* = 1.22). An attrition analysis yielded few differences between the participants who completed the analysis and those who did not (see “Preliminary Analysis”). Table 1 shows the socio-demographic characteristics of the sample.

**Table 1:** Socio-demographic characteristics of the final sample.

Variable	*N*	%
*Gender*		
Male	112	21.3
Female	402	76.4
Other	12	2.3
*Residency*		
Austria	205	39.0
Germany	297	56.5
Switzerland	24	4.6
*First language*		
German	490	93.2
Other	36	6.8
*Current household (Living together)*		
Both parents	375	71.3
Mother	83	15.8
Father	23	4.4
Other^a^	45	8.6
*Current education*		
None	2	0.4
High school	506	96.2
Professional school	14	2.7
Higher education	4	0.8
*Psychotherapy*		
Yes	75	14.3
No	451	85.7
*Physical disability*		
Yes	27	5.1
No	498	94.7
Not answered	1	0.2

a Other current household = Changing family constellations, e.g., spending alternate weeks with each parent.

### Procedure

An anonymous online self-report survey was conducted between December 2020 and May 2021 using the SoSci Survey platform (Leiner, 2019). The average completion time was 23.39 min (*SD* = 7.28). For reimbursement, participants were able to enter a draw to win one of 10 × €15 gift vouchers for an online retailer of their choice (e.g., a fair-trade store, clothing brand, or a general retailer). If desired, they received information about the main results of the study. Participation was voluntary and could be terminated at any given time by closing the browser window. All participants gave explicit informed consent before starting the survey.

### Measures

#### Family cohesion

Family cohesion was assessed using the German version of the Family Adaptability and Cohesion Evaluation Scale IV (FACES IV; Olson, 2011; German version by Stappenbeck et al., 2006), a 42-item self-report instrument assessing flexibility and cohesion in the core family system in line with the Circumplex Model. Due to the curvilinearity of the model, the scale encompasses three 7-item subscales measuring the two extremes and the balanced middle of each dimension: disengagement (e.g., “Family members seem to avoid contact with each other when at home.”), balanced cohesion (e.g., “Our family has a good balance of separateness and closeness.”), and enmeshment (e.g., “We resent family members doing things outside the family.”). For research purposes, a linear ratio score for cohesion and adaptability can be calculated from the three respective subscales of each dimension (Olson, 2011), which we used in the present analysis. In this linear score, family cohesion continually ranges from dysfunctional to functional, with higher scores indicating more functional family cohesion. Items were rated on a 5-point Likert scale (1 = strongly disagree to 5 = strongly agree). In the present study, the internal consistency measured by Cronbach’s α was acceptable for disengagement (0.79) and balanced cohesion (0.87), but low for enmeshment (0.51).

#### Shame- and guilt-proneness

Shame-and guilt-proneness were assessed using the German version of the Test of Self-Conscious Affect for Adolescents (TOSCA-A; Tangney et al., 1991; German version by Kronmüller et al., 2008), a scenario-based self-report instrument for the assessment of dispositional self-conscious emotions. Each of the 15 scenarios (e.g., “You forgot to buy a birthday present.”) is followed by possible reactions (e.g., “I would feel irresponsible and thoughtless.”). Respondents are asked to rate how likely they would be to react in the manner stated on a 5-point Likert scale (1 = not at all likely to 5 = very likely). In the present study, the internal consistency as measured by Cronbach’s α was acceptable for shame-proneness (0.87) and guilt-proneness (0.78).

#### Expressive suppression

Expressive suppression was measured using the Emotion Expression Scale for Children (EESC; Penza-Clyve and Zeman, 2002; German version by Nitkowski et al., 2019). The EESC is a 16-item questionnaire assessing poor emotional awareness with eight items (e.g., “I often do not know why I am angry.”) and expressive reluctance with eight items (e.g., “I don’t show how I really feel in order not to hurt others’ feelings.”). All items are rated on a 5-point scale (1 = not at all true to 5 = extremely true). The original version of the EESC has two dimensions: poor awareness and expressive suppression. However, the German validation study by Nitkowski et al. (2019) did not replicate this finding and the authors thus argue for a unidimensional structure with only 13 of the original items (“Low Emotion Awareness/Suppression”; PA3, PA7, and ES3 excluded). We therefore followed the German recommendation, but first evaluated the fit to our data (see “Preliminary Analyses”). In the present study, Cronbach’s α was acceptable for the full scale (0.89) as well as for expressive reluctance (0.86) and poor awareness (0.82).

#### Internalizing and externalizing problems

Internalizing and externalizing problems were assessed using the German version of the Strengths and Difficulties Questionnaire (SDQ; Goodman, 1997; German version by Lohbeck et al., 2015). The SDQ measures hyperactivity and emotional, behavioral, and peer problems with five items each (e.g., “I am often unhappy, depressed, or tearful.” or “I get very angry and often lose my temper.”). Items are rated on a 3-point rating scale (0 = not true, 1 = somewhat true, and 2 = certainly true). For the purpose of the present study, we used composite scores for internalizing problems (emotional and peer problems) and externalizing problems (behavioral problems and hyperactivity; Goodman et al., 2010). In the present study, Cronbach’s α was acceptable for internalizing problems (0.77) and externalizing problems (0.70).

#### Control variables

Previous findings revealed effects of age and gender on adolescents’ internalizing and externalizing problems, shame-proneness, and emotion regulation (e.g., Zahn-Waxler et al., 2008; Else-Quest et al., 2012; Zimmermann and Iwanski, 2014). We thus added age (in years) and gender (female, male, other; dummy-coded with the reference group female) as control variables to the model. Furthermore, as our measure of shame-proneness (TOSCA-A) shows considerable overlap with the measurement of guilt-proneness, we followed the recommendation to control for guilt-proneness in order to assess “guilt-free shame” (Boudewyns et al., 2013).

### Data analysis

Data were analyzed using SPSS 26 (IBM Corp. Release, 2019) for descriptive statistics and Mplus 8.5 (Muthén and Muthén, 2017) for all structural equation modeling (SEM). Prior to model analysis, we performed an attrition analysis comparing the eligible participants who discontinued the survey with those who completed it. Moreover, as Nitkowski et al. (2019) argued that the German EESC is one-dimensional instead of two-dimensional, we performed a confirmatory factor analysis (CFA) of our data to investigate the data structure. For the path model, we used all variables as mean scores, except for family cohesion, for which a linear ratio score was computed (see above). Missing data were excluded listwise. The model was estimated using robust maximum likelihood estimation (MLR). To evaluate the model fit, we used CFI, TLI, RMSEA, and SRMR, employing the cut-off values reported by Hu and Bentler (1999), with the aim to achieve at least acceptable values on all fit indices (CFI/TLI > 0.90; RMSEA/SRMR <.08). We tested our path model with control variables, by regressing all outcome variables on the control variables.

## Results

### Descriptive statistics

Table 2 provides an overview of the means, standard deviations, and bivariate correlations of the central study variables. Tests and visual inspections for multivariate normality, homoscedasticity, linearity, residual independence, and multicollinearity showed no violations of the assumptions of linear regression.

**Table 2:** Intercorrelations, means, and standard deviations of the final sample.

Variable	COH	SP	ES	INT	EXT	GP	Age
COH	2.10 (1.16)						
SP	**−0.230** ^**^	3.21 (0.75)					
ES	**−0.377** ^**^	**0.555** ^**^	3.18 (0.87)				
INT	**−0.363** ^**^	**0.632** ^**^	**0.642** ^**^	0.94 (0.42)			
EXT	**−0.347** ^**^	**0.214** ^**^	**0.341** ^**^	**0.317** ^**^	0.63 (0.34)		
GP	**0.144** ^**^	**0.458** ^**^	**0.162** ^**^	**0.214** ^**^	−0.052	3.94 (0.51)	
Age	−0.009	−0.058	0.001	0.015	−0.070	0.081	15.74 (1.22)

COH = family cohesion, SP = shame-proneness, ES = expressive suppression, INT = internalizing problems, EXT = externalizing problems, GP = guilt-proneness. Means and standard deviations of the respective variables are displayed on the table diagonal [*M* (*SD*)]. Significant values are in bold.

** *p* < 0.001.

### Preliminary analyses

In total, data from 409 participants (43.7%) of the initial sample (i.e., *N* = 935) were excluded prior to our analysis for different reasons (see “Participants”). The majority of those excluded (*n* = 364, 89%) dropped out voluntarily (i.e., terminated the survey for any reason): Thirteen participants (3.2%) had completely missing datasets, 99 participants (24.2%) dropped out during the first socio-demographic questions, and the remainder (61.6%) dropped out over the course of the study. Patterns of missing data in the analysis revealed a gradual rise in missing data parallel to the order of questionnaires in the study, probably due to the survey length. Comparing the non-completers to the valid completers in terms of socio-demographic variables, it emerged that non-completers had a higher proportion of male or other gender rather than female gender [*χ^2^*(2) = 13.495, *p* = 0.001]. Moreover, a higher proportion of non-completers reported to be of German and Swiss residency compared to Austrian residency [*χ^2^*(3) = 16.263, *p* = 0.001]. Further, non-completers (*M* = 3.79, *SD* = 1.11) lived in smaller households compared to completers [*M* = 4.06, *SD* = 1.16, *t*(860) = 3.373, *p* = 0.001]. Non-completers (*M* = 1.87, *SD* = 1.00) also lived with less children in the household than completers [*M* = 2.02, *SD* = 0.97, *t*(860) = 2.281, *p* = 0.023], although the percentage of children in the household did not differ between groups [*t*(855) = 1.224, *p* = 0.221]. The means and distributions of all other socio-demographic variables did not differ between the two groups.

Using the final sample of 526 adolescents only, we ran a CFA for the EESC in order to test its factorial structure. We tested both (1) a 13-item one-factor model (German validation) and (2) a 16-item two-factor model (English original). Initially, neither model yielded an acceptable model fit (Model 1: CFI = 0.771, TLI = 0.725, RMSEA = 0.130, SRMR = 0.073; Model 2: CFI = 0.836, TLI = 0.809, RMSEA = 0.097, SRMR = 0.067). However, when specifying covariances between items based on content and linguistic similarity (PA2 with PA5, ES1 with ES2, ES4 with ES7, ES4 with PA4, ES5 with ES8), both models yielded an acceptable fit (Model 1: CFI = 0.923, TLI = 0.900, RMSEA = 0.078, SRMR = 0.048; Model 2: CFI = 0.934, TLI = 0.919, RMSEA = 0.063, SRMR = 0.049). Due to the similarity of our sample to that used in the German validation study, we used the 13-item single factor as a mean score in the path model.

### Mediation model

**Figure 2:**
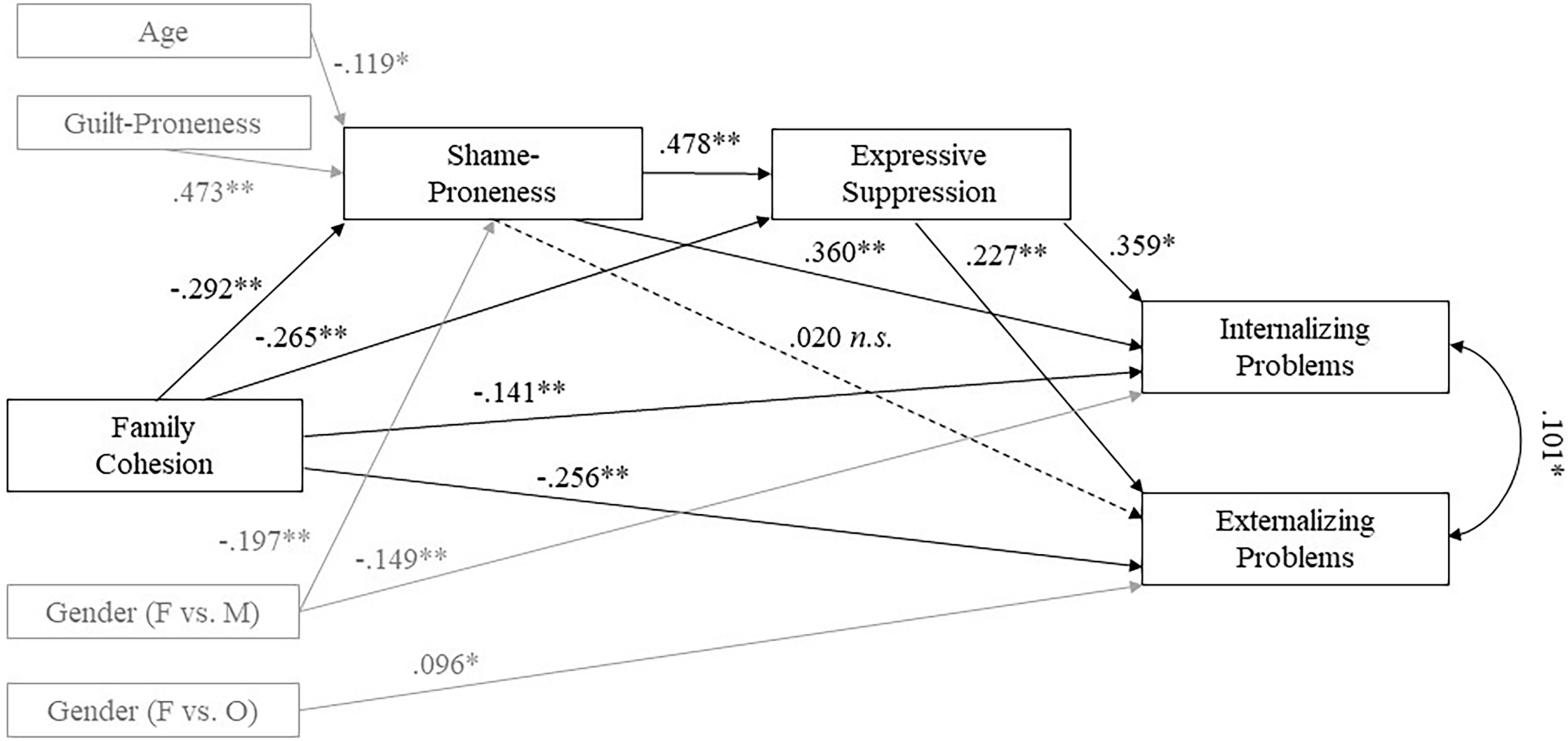
Statistical model with standardized path coefficients. Standardized path coefficients are shown. Control variables and their significant paths are displayed in gray; non-significant paths of the control variables are not displayed. ^*^p < 0.05; ^**^p < 0.001.

We tested the dual mediation model by regressing internalizing and externalizing problems on family cohesion, shame-proneness and expressive suppression, and by regressing shame-proneness and expressive suppression on family cohesion. Additionally, expressive suppression was regressed on shame-proneness. We further regressed all endogenous variables on the control variables age and gender (two dummy-coded variables), and additionally regressed shame-proneness on guilt-proneness in order to assess “guilt-free” shame-proneness, as recommended by Boudewyns et al. (2013). The model fit was acceptable [*χ^2^*(26) = 976.019, *p* < 0.001; CFI = 0.999; TLI = 0.990; RMSEA = 0.027 [90% CI: 0.000–0.083, *p* = 0.495]; SRMR = 0.014]. All paths between the variables were significant in the predicted directions, expect for the non-significant path of shame-proneness to externalizing problems. With regard to the control variables, age and male gender were negatively associated with shame-proneness, and guilt-proneness was positively associated with shame-proneness. Moreover, male gender was negatively related to internalizing problems, and other gender was positively related to externalizing problems. The final model with standardized path estimates is shown in Figure 2 and all standardized and unstandardized regression coefficients for the models are listed in Table 3.

**Table 3:** Direct effects of the path model.

Direct effects	*b* (*SE*)		95% CI	*p*	β (*SE*)		95% CI	*p*	*R^2^*
*Internalizing problems*									0.558
COH	**−0.051**	(0.011)	[−0.072; −0.029]	< 0.001	**−0.141**	(0.031)	[−0.202; −0.080]	< 0.001	
SP	**0.200**	(0.021)	[0.159; 0.241]	< 0.001	**0.360**	(0.038)	[0.287; 0.434]	< 0.001	
ES	**0.172**	(0.018)	[0.136; 0.207]	< 0.001	**0.359**	(0.038)	[0.285; 0.434]	< 0.001	
Age	0.006	(0.010)	[−0.014; 0.026]	0.576	0.017	(0.030)	[−0.042; 0.076]	0.576	
Gender (F vs. M)	**−0.151**	(0.029)	[−0.209; −0.094]	< 0.001	**−0.149**	(0.029)	[−0.205; −0.092]	< 0.001	
Gender (F vs. O)	0.010	(0.061)	[−0.109; 0.130]	0.863	0.004	(0.022)	[−0.039; 0.046]	0.863	
*Externalizing problems*									0.186
COH	**−0.074**	(0.012)	[−0.098; −0.050]	< 0.001	**−0.256**	(0.042)	[−0.337; −0.174]	< 0.001	
SP	0.009	(0.021)	[−0.033; 0.051]	0.675	0.020	(0.048)	[−0.074; 0.115]	0.675	
ES	**0.087**	(0.020)	[0.049; 0.126]	< 0.001	**0.227**	(0.051)	[0.128; 0.327]	< 0.001	
Age	−0.017	(0.011)	[−0.039; 0.005]	0.134	−0.061	(0.041)	[−0.141; 0.019]	0.133	
Gender (F vs. M)	0.001	(0.033)	[−0.065; 0.064]	0.981	−0.001	(0.040)	[−0.080; 0.078]	0.981	
Gender (F vs. O)	**0.215**	(0.083)	[0.052; 0.379]	0.010	**0.096**	(0.039)	[0.019; 0.173]	0.015	
*Expressive suppression*									0.378
COH	**−0.200**	(0.028)	[−0.254; −0.146]	< 0.001	**−0.265**	(0.036)	[−0.336; −0.195]	< 0.001	
SP	**0.555**	(0.042)	[0.473; 0.638]	< 0.001	**0.478**	(0.035)	[0.410; 0.547]	< 0.001	
Age	0.019	(0.025)	[−0.030; 0.068]	0.450	0.027	(0.035)	[−0.043; 0.096]	0.450	
Gender (F vs. M)	−0.112	(0.080)	[−0.269; 0.045]	0.161	−0.053	(0.037)	[−0.126; 0.021]	0.169	
Gender (F vs. O)	0.236	(0.212)	[−0.179; 0.651]	0.264	0.040	(0.037)	[−0.031; 0.112]	0.270	
*Shame-proneness*									0.345
COH	**−0.189**	(0.026)	[−0.240; −0.139]	< 0.001	**−0.292**	(0.039)	[−0.369; −0.215]	< 0.001	
GP	**0.697**	(0.051)	[0.597; 0.798]	< 0.001	**0.473**	(0.033)	[0.407; 0.539]	< 0.001	
Age	**−0.073**	(0.022)	[−0.117; −0.030]	0.001	**−0.119**	(0.036)	[−0.191; −0.048]	0.001	
Gender (F vs. M)	**−0.361**	(0.197)	[−0.482; −0.240]	< 0.001	**−0.197**	(0.034)	[−0.263; −0.130]	< 0.001	
Gender (F vs. O)	0.051	(0.062)	[−0.336; 0.438]	0.796	0.010	(0.039)	[−0.067; 0.087]	0.796	

*b* (*SE*) = unstandardized predictor and standard error, β (*SE*) = standardized predictor and standard error, COH = family cohesion, SP = shame-proneness, GP = guilt-proneness, ES = expressive suppression. Gender was dummy-coded with the reference group “female”: F vs. M = female vs. male, F vs. O = female vs. other gender. Significant values are in bold.

Besides the direct effects, our analysis indicated significant indirect effects from family cohesion to both mental health outcomes (see Table 4). Accordingly, family cohesion was significantly negatively related to internalizing problems via shame-proneness and via expressive suppression as independent single mediators, and via both variables in the dual mediation model. For externalizing problems as outcome variable, the mediation through both mediators as well as through expressive suppression as a single mediator was significant, but mediation through shame-proneness alone was not significant. The total indirect effect was higher for internalizing than for externalizing problems [Δβ = 0.153; χ^2^(1) = 36.106, *p* < 0.001], and this also applied to the specific indirect effects [both mediators: Δβ = 0.018, χ^2^(1) = 8.337, *p* = 0.004; single mediator SP: Δβ = 0.099; χ^2^(1) = 24.552, *p* < 0.001; single mediator ES: Δβ = 0.035; χ^2^(1) = 8.976; *p* = 0.003]. The standardized and unstandardized indirect effects are listed in Table 4.

**Table 4:** Indirect effects of the path model.

Total and indirect effects	*b* (*SE*)		95% CI	β (*SE*)		95% CI	*p*
*Internalizing problems*							
Total effect	**−0.141**	(0.014)	[−0.167; −0.114]	**−0.391**	(0.037)	[−0.463; −0.319]	<0.001
Total indirect effect	**−0.090**	(0.011)	[−0.111; −0.069]	**−0.251**	(0.028)	[−0.306; −0.195]	<0.001
1. COH → SP → INT	**−0.038**	(0.006)	[−0.050; −0.025]	**−0.105**	(0.017)	[−0.139; −0.072]	<0.001
2. COH → ES → INT	**−0.034**	(0.006)	[−0.046; −0.022]	**−0.095**	(0.017)	[−0.128; −0.063]	<0.001
3. COH → SP → ES → INT	**−0.018**	(0.003)	[−0.025; −0.011]	**−0.050**	(0.009)	[−0.068; −0.032]	<0.001
*Externalizing problems*							
Total effect	**−0.102**	(0.012)	[−0.125; −0.080]	**−0.354**	(0.038)	[−0.429; −0.279]	<0.001
Total indirect effect	**−0.028**	(0.006)	[−0.040; −0.017]	**−0.098**	(0.020)	[−0.136; −0.060]	<0.001
4. COH → SP → EXT	−0.002	(0.004)	[−0.010; 0.006]	−0.006	(0.014)	[−0.033; 0.022]	0.674
5. COH → ES → EXT	**−0.017**	(0.005)	[−0.026; −0.008]	**−0.060**	(0.016)	[−0.091; −0.030]	<0.001
6. COH → SP → ES → EXT	**−0.009**	(0.002)	[−0.014; −0.005]	**−0.032**	(0.008)	[−0.048; −0.016]	<0.001

*b* (*SE*) = unstandardized predictor and standard error, β (*SE*) = standardized predictor and standard error, COH = family cohesion, SP = shame-proneness, ES = expressive suppression, INT = internalizing problems, EXT = externalizing problems. Significant values are in bold.

The final model was able to account for 56% of the variance in internalizing problems and 19% of the variance in externalizing problems. It further explained 38.7% of the variance in expressive suppression, and 34.5% in shame-proneness. However, without guilt-proneness, only 13.4% of shame-proneness was explained by family cohesion.

### Additional analyses

A conceptual revision of the TOSCA-A items revealed two morally ambivalent items on the shame-proneness subscale (items 7 and 12), in which the shame reaction is not entirely maladaptive but may be morally relevant. A *post-hoc* analysis under the omission of those two items improved the model fit and resulted in stronger path coefficients. We, however, base our interpretation on the previous model using the full TOSCA-A. We further tested our theory by analyzing the same model again with guilt-proneness instead of shame-proneness as the mediator, and analogously partialized shame out of the outcomes to assess shame-free guilt. This analysis revealed that family cohesion was positively related to guilt-proneness, but the remaining pathways to psychopathology via guilt-proneness were not significant. In sum, these re-analyses speak for the robustness of our empirical model and buttress our theoretical and conceptual assumptions. The results of the additional analyses are presented in the Supplementary Tables S1–S5.

## Discussion

The family is a crucial context for adolescents’ development and well-being. Therefore, we developed our model to shed light on the interrelations between family cohesion, emotional experiences, and mental health problems in adolescents. The existing literature strongly supports the notion that a dysfunctional family environment is associated with adolescents’ internalizing and externalizing problems (Hughes and Gullone, 2008; Pinquart, 2017). We hypothesized that one possible pathway through which this effect occurs is a disposition to feel ashamed and an overreliance on expressive suppression. Both shame-proneness and expressive suppression have been found to be associated with a plethora of negative mental health outcomes in children, adolescents, and adults (Tangney et al., 1992; Trosper et al., 2009; Gross and Cassidy, 2019). Recent studies have provided further evidence that shame and expressive suppression can mediate the relationship between parenting practices and mental health problems (Mills, 2005; Balan et al., 2017). We were able to confirm the majority of our hypotheses derived from this earlier literature. Specifically, we found that expressive suppression and shame-proneness mediated the effects of family cohesion on internalizing problems in adolescents, both separately and in the form of a dual mediation. For externalizing problems, we found a dual mediation and a single mediation through expressive suppression but not through shame-proneness. Moreover, there was no significant association between shame-proneness and adolescents’ externalizing problems. In conclusion, our model explained 56% of the variance in internalizing problems, but only 19% of the variance in externalizing problems. We further found significant effects of the control variables age and gender on shame-proneness and mental health problems, with the direction of the path coefficients being in line with earlier research (Zahn-Waxler et al., 2008; Else-Quest et al., 2012).

The pattern of associations with mental health problems found in the present study may be indicative of the differential effects of shame on psychological adjustment and on the regulation of challenging emotions. According to the compass of shame (Elison et al., 2006a), the consequences of state shame can be either externalizing or internalizing: On the one hand, shame is a self-directed emotion and thus inward, causing one to feel unworthy and defective. The strong associations between shame and internalizing problems confirm this inward-looking tendency (Kim et al., 2011; Cândea and Szentagotai-Tătar, 2018). On the other hand, however, the consequences of shame can also reveal themselves externally, for instance by blaming other people or by behaving hostilely and aggressively (Tangney et al., 1992; Elison et al., 2006a). In our model, shame-proneness was not linked to externalizing problems per se, but a significant indirect effect emerged when considering expressive suppression as a mediator. Thus, it is possible that suppression of negative affect alone leads to negative behavioral outcomes, whereas shame alone leads mainly to negative emotional outcomes. This fits well with the concept of shame acknowledgment within the compass of shame (Elison et al., 2006a): When attacking the self or withdrawing after a shameful experience, the experience is acknowledged as negative, although not necessarily perceived as shame. The externalizing consequences, however, may not involve acknowledgment of shame or at least may not involve an acceptance of the negative feelings. Instead, the negative feelings may be made unconscious and replaced by other behaviors, or they may elicit anger, which is then directed outwards toward others. Thus, expressive suppression appears to be a plausible connector between shame and its psychological outcomes. Nevertheless, this explanation might not be exhaustive in view of the low amount of explained variance. Further, it should be noted that our measure to assess internalizing problems includes peer problems (example items: “Other people pick on me or bully me”; “Other people generally like me” [recoded]). Given the interpersonal function of shame, we believe that the negative consequences of shame are particularly pronounced in relationships with others. However, the behavioral consequences of shame may also include non-interpersonal aggression or distraction behavior (Elison et al., 2006b). Thus, the lack of a significant direct relationship between shame-proneness and externalizing problems cannot be attributed solely to the methodology of the present study.

As described earlier, past research showed that shame is often associated with psychological maladjustment, especially at the dispositional level. Guilt, on the other hand, involves more behavior-specific attributions and may lead to more adaptive reactions. The distinction between shame and guilt in maladaptive versus adaptive might, however, be too reductive. Thus, according to Dempsey (2017), context and response should also be examined. We accommodate this idea by including emotion regulation in our analyses. Moreover, since the TOSCA-A is particularly suited for examining maladaptive shame and adaptive guilt (including behavioral consequences; Luyten et al., 2002), we related our rationale to the maladaptive sides of shame. Our control analyses with unambiguously maladaptive shame-proneness (excluding items 7 and 12 of the TOSCA-A) and guilt-proneness as a mediator supported this view (see Supplementary Tables S1–S5).

Family science is complex and manifold, and family relationships can be investigated at different levels of specificity, from the bilateral relationship between two individuals to the global, systemic level (Watson, 2012). Family systems theory (FST) has received growing attention from developmental and clinical psychologists in the past few decades (Cox and Paley, 2003), and argues for a comprehensive view that includes the various relationships in family dynamics. Such a broader systemic view allowed us to look at the web of relationships within the family; however, it seems that this may blur information about the more specific aspects in this realm. Scholars often investigate specific dimensions of family relationships (e.g., interparental conflict, parenting behavior) that may lead to mental health problems, shame, and emotional dysregulation among children and adolescents (Mills, 2005; Balan et al., 2017; van Dijk et al., 2020). That being said, the systemic level is by no means independent of the more specific aspects of family life, as these represent experiences in the subsystems (Cox and Paley, 2003). The overall systemic view might further help to foster our understanding of certain behaviors, such as the adaptivity of emotion regulation strategies. For instance, in a disengaged family environment, suppressing or avoiding intense emotions can be an adaptive way of dealing with emotional arousal, given that openly expressed emotions might be ignored or even punished (Repetti et al., 2002; Southam-Gerow and Kendall, 2002). Shame is further considered to be a social emotion, which according to evolutionary psychologists is used to establish social hierarchies (Gilbert, 2000). It is thus possible that in accordance with FST, shame serves the function of establishing and maintaining the family hierarchy and homeostasis (Loader, 1998).

Multigenerational, single parent, co-parenting, and rainbow families represent specific developmental contexts for adolescents. Since we did not ask our participants about the gender, sexual orientation, or sexual identity of their parents, we cannot draw conclusions for the latter. However, we believe that our model is also applicable to non-heterosexual and/or non-cisgender families, as the FACES IV assessed global systemic levels of cohesion within the family without explicitly naming a specific family member (e.g., father or mother), only referring to “our family.” Moreover, children of LGBTQ+ parents show similar psychological adjustment compared to those raised in heterosexual, cisgender families (e.g., Fedewa et al., 2015). Thus, intrafamily processes may be of greater interest than family sexual orientation or identity per se (Goldberg and Sweeney, 2019). Single and divorced parenting has been associated with poorer adaption in children (Daryanani et al., 2016; van Dijk et al., 2020). Nevertheless, again, intrafamily processes have been shown to be more relevant for our primary study variables than family structure or marital status per se (Walter and Burnaford, 2006; Shaffer et al., 2012; van Dijk et al., 2020), making our model applicable to a variety of family forms. Emotional closeness and self-regulation are further intergenerationally transmitted, which would call for further evaluation of the multigenerational family context (Bridgett et al., 2015; Hank et al., 2017).

Further aspects of our results need to be interpreted with caution. Although we analyzed our constructs in a linear manner, it is important to consider that they likely interact with each other and thus exhibit bidirectional associations. For instance, mental health problems are often stigmatized and might thus elicit shame and avoidant coping in adolescents (Schibalski et al., 2017; Ferrie et al., 2020). Youth emotional or conduct problems can also negatively influence the family climate (Steeger and Gondoli, 2013). This is particularly valid when viewed through the lens of the FST framework, in which the family is regarded as an organized unit and its elements or subsystems are inextricably interconnected; thus, there is a reciprocal transfer of mood, affect, or behaviors across family subsystems and members. Our model could also benefit from being tested longitudinally, preferably involving more than one family member. The developmental components would require longitudinal testing over several months or years. The constructs could also influence each other over a shorter period, perhaps on a daily basis, for which intensive longitudinal data would be needed (e.g., an EMA study; Shiffman et al., 2008). The inclusion of a clinical assessment of family functioning or psychological problems (by expert ratings) could further increase the validity of the measurements. Additionally, given our model has been purely correlational to date, family-level interventions (in this case to strengthen family cohesion, for example) may hold promise for examining causality in the model (Cowan and Cowan, 2002).

Furthermore, there may be other variables that act as mediators on the pathways we investigated, such as self-esteem (Leung et al., 1996; Guo et al., 2018), loneliness (Wang et al., 2020), and emotional distress (Soloski and Berryhill, 2016). Notably, all of these potential mediators represent intra-psychic, negative self-related emotions or might be related to expressive suppression. As such, it is possible that the inclusion of these aspects could either leverage our model, or could fade out our proposed mediators in view of the inconsistent terminology and conceptual fuzziness of these concepts.

Family relationships remain hugely important for individual development into adulthood. Nevertheless, it is evident that the family loses importance during adolescence in favor of peer relationships (Smetana et al., 2014), and shame may arise more easily as social comparisons become more central (Gilbert and Irons, 2008). Expressive suppression may mediate the link between negative experiences in the peer context and heightened feelings of loneliness as well as internalizing or externalizing problems (Gardner et al., 2017; Herd and Kim-Spoon, 2021). Future studies might additionally consider the peer context and general fears of negative evaluation (Teachman and Allen, 2007) in the development of shame-proneness, emotion regulation, and mental well-being.

### Limitations

The first and major limitation of our study lies in its cross-sectional design. Mediation implies temporal sequences of the concepts; thus, applying mediation analysis to cross-sectional data might generate bias (Maxwell et al., 2011). Although we theoretically derived the direction of effects and mediating mechanisms, we cannot infer causal relationships.

Second, the exclusive reliance on adolescents’ perspective by means of self-report provides a limited proxy for assessing family cohesion and youth mental health, and effects may therefore be inflated because of common-method variance. To address this issue, future studies could incorporate multiple family members’ perspectives and consider multi-method approaches.

Third, it is important to note that the measure used to assess family cohesion in the present study, the FACES IV, does not differentiate between the specific directions of family dysfunction. As the family cohesion ratio scale from the FACES IV was developed for the purpose of linear modeling in research (Olson, 2011), the questionnaire is not recommended for the assessment of disengagement and enmeshment separately for research purposes. However, combining two qualitatively different constructs for methodological reasons, although considered equally maladaptive, might be less informative than measuring them separately.

Forth, shame and emotional expression as well as family systems are influenced by the cultural context (Butler et al., 2007; Wong and Tsai, 2007); our results can only be applied to the middle European context.

Lastly, it is important to note that this study was conducted during the COVID-19 pandemic. During this time, the mental health of young people has been jeopardized, as reflected in the increasing prevalence rates of mental health problems among children and adolescents in German-speaking countries (Ravens-Sieberer et al., 2021). Young people’s resources are depleted by school closures and other disruptions to public life (Lee, 2020). Close family living might also be subject to novel tensions, which might in turn strain family relationships (Calvano et al., 2021), although conversely, family support may also buffer pandemic-related stress (van Eickels et al., 2022). In conclusion, we cannot rule out the possibility that our findings were influenced by the pandemic situation.

## Conclusion

We tested a comprehensive model of potential pathways from the emotional relationships in the family to mental health problems in adolescents. Our results indicate that family cohesion may play a role in adolescents’ predisposition to feelings of shame and difficulties in expressing their emotions, which are in turn linked to more internalizing and externalizing problems. The high prevalence of mental health problems in adolescents in the German-speaking countries underlines the importance of a thorough understanding of their underlying processes, and our results emphasize the importance of considering the family system in this context.

## Data availability statement

The datasets presented in this study can be found in online repositories. The names of the repository/repositories and accession number(s) can be found at: https://osf.io/tp68r/.

## Ethics statement

The studies involving human participants were reviewed and approved by the institutional review board of the University of Vienna, Vienna, Austria (Reference number: 00596; Date of approval: 24.11.2020). Written informed consent from the participants’ legal guardian/next of kin was not required to participate in this study in accordance with the national legislation and the institutional requirements.

## Author contributions

RE planned and directed the study, collected the data, performed the analysis, and drafted the manuscript. AT-F assisted with the main analysis, drafted parts of the manuscript, and revised the manuscript. MZ supervised the study procedures and data analysis, provided theoretical input, and revised the manuscript. All authors contributed to the article and approved the submitted version.

## Conflict of interest

The authors declare that the research was conducted in the absence of any commercial or financial relationships that could be construed as a potential conflict of interest.

## Publisher’s note

All claims expressed in this article are solely those of the authors and do not necessarily represent those of their affiliated organizations, or those of the publisher, the editors and the reviewers. Any product that may be evaluated in this article, or claim that may be made by its manufacturer, is not guaranteed or endorsed by the publisher.

## Supplementary material

The Supplementary material for this article can be found online at: https://www.frontiersin.org/articles/10.3389/fpsyg. 2022.921250/full#supplementary-material

## References

[ref1] Abell E. Gecas V. (1997). Guilt, shame, and family socialization: a retrospective study. J. Fam. Issues 18, 99–123. doi: 10.1177/019251397018002001

[ref2] Balan R. Dobrean A. Roman G. D. Balazsi R. (2017). Indirect effects of parenting practices on internalizing problems among adolescents: the role of expressive suppression. J. Child Fam. Stud. 26, 40–47. doi: 10.1007/s10826-016-0532-4

[ref3] Barber B. K. Buehler C. (1996). Family cohesion and enmeshment: different constructs, different effects. J. Marriage Fam. 58:433. doi: 10.2307/353507

[ref4] Bariola E. Hughes E. K. Gullone E. (2012). Relationships between parent and child emotion regulation strategy use: a brief report. J. Child Fam. Stud. 21, 443–448. doi: 10.1007/s10826-011-9497-5

[ref5] Bennett D. S. Sullivan M. W. Lewis M. (2010). Neglected children, shame-proneness, and depressive symptoms. Child Maltreat. 15, 305–314. doi: 10.1177/1077559510379634, PMID: 20724372 PMC3771652

[ref6] Boudewyns V. Turner M. M. Paquin R. S. (2013). Shame-free guilt appeals: testing the emotional and cognitive effects of shame and guilt appeals. Psychol. Mark. 30, 811–825. doi: 10.1002/mar.20647

[ref7] Bridgett D. J. Burt N. M. Edwards E. S. Deater-Deckard K. (2015). Intergenerational transmission of self-regulation: a multidisciplinary review and integrative conceptual framework. Psychol. Bull. 141, 602–654. doi: 10.1037/a0038662, PMID: 25938878 PMC4422221

[ref8] Buchman-Wildbaum T. Unoka Z. Dudas R. Vizin G. Demetrovics Z. Richman M. J. (2021). Shame in borderline personality disorder: meta-analysis. J. Personal. Disord. 35, 149–161. doi: 10.1521/pedi_2021_35_515, PMID: 33650893

[ref9] Butler E. A. Lee T. L. Gross J. J. (2007). Emotion regulation and culture: are the social consequences of emotion suppression culture-specific? Emotion 7, 30–48. doi: 10.1037/1528-3542.7.1.30, PMID: 17352561

[ref10] Calvano C. Engelke L. Di Bella J. Kindermann J. Renneberg B. Winter S. M. (2021). Families in the COVID-19 pandemic: parental stress, parent mental health and the occurrence of adverse childhood experiences—results of a representative survey in Germany. Eur. Child Adolesc. Psychiatry. doi: 10.1007/s00787-021-01739-0 [Epub ahead of print]., PMID: 33646416 PMC7917379

[ref11] Campbell-Sills L. Ellard K. K. Barlow D. H. (2014). “Emotion regulation in anxiety disorders,” in Handbook of Emotion Regulation. ed. Grooss J. J. (New York, NY, United States: Guilford Publications), 393–412.

[ref12] Cândea D.-M. Szentagotai-Tătar A. (2018). Shame-proneness, guilt-proneness and anxiety symptoms: a meta-analysis. J. Anxiety Disord. 58, 78–106. doi: 10.1016/j.janxdis.2018.07.005, PMID: 30075356

[ref13] Carpenter T. P. Tignor S. M. Tsang J.-A. Willett A. (2016). Dispositional self-forgiveness, guilt-and shame-proneness, and the roles of motivational tendencies. Personal. Individ. Differ. 98, 53–61. doi: 10.1016/j.paid.2016.04.017

[ref14] Cerniglia L. Cimino S. Tafà M. Marzilli E. Ballarotto G. Bracaglia F. (2017). Family profiles in eating disorders: family functioning and psychopathology. Psychol. Res. Behav. Manag. 10, 305–312. doi: 10.2147/PRBM.S145463 29042824 PMC5633277

[ref15] Cesare C. Francesco P. Valentino Z. Barbara D. Olivia R. Gianluca C.. (2016). Shame proneness and eating disorders: a comparison between clinical and non-clinical samples. Eat. Weight Disord. - Stud. Anorex. Bulim. Obes 21, 701–707. doi: 10.1007/s40519-016-0328-y, PMID: 27704341

[ref16] Chen E. Brody G. H. Miller G. E. (2017). Childhood close family relationships and health. Am. Psychol. 72, 555–566. doi: 10.1037/amp0000067, PMID: 28880102 PMC5598786

[ref17] Coll K. M. Juhnke G. A. Thobro P. Haas R. Smith Robinson M. (2008). Family disengagement of youth offenders: implications for counselors. Fam. J. 16, 359–363. doi: 10.1177/1066480708322805

[ref18] Cowan P. A. Cowan C. P. (2002). Interventions as tests of family systems theories: marital and family relationships in children’s development and psychopathology. Dev. Psychopathol. 14, 731–759. doi: 10.1017/S0954579402004054, PMID: 12549702

[ref19] Cox M. J. Paley B. (2003). Understanding families as systems. Curr. Dir. Psychol. Sci. 12, 193–196. doi: 10.1111/1467-8721.01259, PMID: 35848616

[ref20] Crane J. Harper J. M. Bean R. A. Holmes E. (2020). Family implicit rules, shame, and adolescent prosocial and antisocial communication behaviors. Fam. J. 28, 72–82. doi: 10.1177/1066480719896563

[ref21] Daryanani I. Hamilton J. L. Abramson L. Y. Alloy L. B. (2016). Single mother parenting and adolescent psychopathology. J. Abnorm. Child Psychol. 44, 1411–1423. doi: 10.1007/s10802-016-0128-x, PMID: 26767832 PMC5226056

[ref22] de Hooge I. E. Breugelmans S. M. Zeelenberg M. (2008). Not so ugly after all: when shame acts as a commitment device. J. Pers. Soc. Psychol. 95, 933–943. doi: 10.1037/a0011991, PMID: 18808269

[ref23] de Hooge I. E. Zeelenberg M. Breugelmans S. M. (2010). Restore and protect motivations following shame. Cognit. Emot. 24, 111–127. doi: 10.1080/02699930802584466, PMID: 21824031

[ref24] Dearing R. L. Stuewig J. Tangney J. P. (2005). On the importance of distinguishing shame from guilt: Relations to problematic alcohol and drug use. Addict. Behav. 30, 1392–1404. doi: 10.1016/j.addbeh.2005.02.002, PMID: 16022935 PMC3106346

[ref25] Dempsey H. L. (2017). A Comparison of the Social-Adaptive Perspective and Functionalist Perspective on Guilt and Shame. Behav. Sci. 7:83. doi: 10.3390/bs7040083, PMID: 29232888 PMC5746692

[ref26] Dryman M. T. Heimberg R. G. (2018). Emotion regulation in social anxiety and depression: a systematic review of expressive suppression and cognitive reappraisal. Clin. Psychol. Rev. 65, 17–42. doi: 10.1016/j.cpr.2018.07.004, PMID: 30064053

[ref27] Eisenberg N. (1996). Meta-emotion and socialization of emotion in the family--A topic whose time has come: Comment on Gottman et al. (1996). J. Fam. Psychol. 10, 269–276. doi: 10.1037/0893-3200.10.3.269

[ref28] Elison J. Lennon R. Pulos S. (2006a). Investigating the compass of shame: the development of the compass of shame scale. Soc. Behav. Personal. Int. J. 34, 221–238. doi: 10.2224/sbp.2006.34.3.221

[ref29] Elison J. Pulos S. Lennon R. (2006b). Shame-focused coping: an empirical study of the compass of shame. Soc. Behav. Personal. Int. J. 34, 161–168. doi: 10.2224/sbp.2006.34.2.161

[ref30] Else-Quest N. M. Higgins A. Allison C. Morton L. C. (2012). Gender differences in self-conscious emotional experience: a meta-analysis. Psychol. Bull. 138, 947–981. doi: 10.1037/a0027930, PMID: 22468881

[ref31] Eschmann S. Weber Häner Y. Steinhausen H.-C. (2007). Die Prävalenz psychischer Störungen bei Kindern und Jugendlichen unter Berücksichtigung soziodemografischer Merkmale [The prevalence of mental disorders in children and adolescents considering sociodemographic characteristics]. Z. Für Klin. Psychol. Psychother. 36, 270–279. doi: 10.1026/1616-3443.36.4.270

[ref32] Fedewa A. L. Black W. W. Ahn S. (2015). Children and adolescents with same-gender parents: a meta-analytic approach in assessing outcomes. J. GLBT Fam. Stud. 11, 1–34. doi: 10.1080/1550428X.2013.869486

[ref33] Ferrie J. Miller H. Hunter S. C. (2020). Psychosocial outcomes of mental illness stigma in children and adolescents: a mixed-methods systematic review. Child Youth Serv. Rev. 113:4961. doi: 10.1016/j.childyouth.2020.104961

[ref34] Fosco G. M. Grych J. H. (2013). Capturing the family context of emotion regulation: a family systems model comparison approach. J. Fam. Issues 34, 557–578. doi: 10.1177/0192513X12445889

[ref35] Fox N. A. Calkins S. D. (2003). The development of self-control of emotion: intrinsic and extrinsic influences. Motiv. Emot. 27, 7–26. doi: 10.1023/A:1023622324898, PMID: 30869939

[ref36] Fuchs M. Karwautz A. (2017). Epidemiologie psychischer Störungen bei Kindern und Jugendlichen: Eine narrative Übersichtsarbeit unter Berücksichtigung österreichischer Daten [Epidemiology of mental disorders in children and adolescents: a narrative review considering austrian data]. Neuropsychiatrie 31, 96–102. doi: 10.1007/s40211-017-0238-x 28853032

[ref37] Gardner S. E. Betts L. R. Stiller J. Coates J. (2017). The role of emotion regulation for coping with school-based peer-victimisation in late childhood. Personal. Individ. Differ. 107, 108–113. doi: 10.1016/j.paid.2016.11.035

[ref38] Gausel N. Vignoles V. L. Leach C. W. (2016). Resolving the paradox of shame: differentiating among specific appraisal-feeling combinations explains pro-social and self-defensive motivation. Motiv. Emot. 40, 118–139. doi: 10.1007/s11031-015-9513-y

[ref39] Gilbert P. (2000). The relationship of shame, social anxiety and depression: the role of the evaluation of social rank. Clin. Psychol. Psychother. 7, 174–189. doi: 10.1002/1099-0879(200007)7:3<174::AID-CPP236>3.0.CO;2-U

[ref40] Gilbert P. Irons C. (2008). “Shame, self-criticism, and self-compassion in adolescence,” in Adolescent Emotional Development and the Emergence of Depressive Disorders. eds. Allen N. B. Sheeber L. B. (Cambridge: Cambridge University Press), 195–214.

[ref41] Goldberg A. E. Sweeney K. K. (2019). “LGBTQ parent families,” in APA Handbook of Contemporary Family Psychology: Foundations, Methods, and Contemporary Issues Across the Lifespan. Vol. 1 eds. Fiese B. H. Celano M. Deater-Deckard K. Jouriles E. N. Whisman M. A. (Washington: American Psychological Association), 743–760.

[ref42] Goodman R. (1997). The strengths and difficulties questionnaire: a research note. J. Child Psychol. Psychiatry 38, 581–586. doi: 10.1111/j.1469-7610.1997.tb01545.x, PMID: 9255702

[ref43] Goodman A. Lamping D. L. Ploubidis G. B. (2010). When to use broader internalising and externalising subscales instead of the hypothesised five subscales on the strengths and difficulties questionnaire (SDQ): data from british parents, teachers and children. J. Abnorm. Child Psychol. 38, 1179–1191. doi: 10.1007/s10802-010-9434-x, PMID: 20623175

[ref44] Gross J. T. Cassidy J. (2019). Expressive suppression of negative emotions in children and adolescents: theory, data, and a guide for future research. Dev. Psychol. 55, 1938–1950. doi: 10.1037/dev0000722, PMID: 31464496

[ref45] Gross C. A. Hansen N. E. (2000). Clarifying the experience of shame: the role of attachment style, gender, and investment in relatedness. Personal. Individ. Differ. 28, 897–907. doi: 10.1016/S0191-8869(99)00148-8

[ref46] Gross J. J. John O. P. (2003). Individual differences in two emotion regulation processes: implications for affect, relationships, and well-being. J. Pers. Soc. Psychol. 85, 348–362. doi: 10.1037/0022-3514.85.2.348, PMID: 12916575

[ref47] Guo L. Tian L. Scott Huebner E. (2018). Family dysfunction and anxiety in adolescents: a moderated mediation model of self-esteem and perceived school stress. J. Sch. Psychol. 69, 16–27. doi: 10.1016/j.jsp.2018.04.002, PMID: 30558751

[ref48] Gupta S. Zachary Rosenthal M. Mancini A. D. Cheavens J. S. Lynch T. R. (2008). Emotion regulation skills mediate the effects of shame on eating disorder symptoms in women. Eat. Disord. 16, 405–417. doi: 10.1080/10640260802370572, PMID: 18821364

[ref49] Hank K. Salzburger V. Silverstein M. (2017). Intergenerational transmission of parent-child relationship quality: evidence from a multi-actor survey. Soc. Sci. Res. 67, 129–137. doi: 10.1016/j.ssresearch.2017.06.004, PMID: 28888280

[ref50] Harter S. (2006). “The self,” in Handbook of Child Psychology: Social, Emotional, and Personality Development. eds. Eisenberg N. Damon W. Lerner R. M. (Hoboken, NJ, United States: John Wiley & Sons Inc.), 505–570.

[ref51] Herd T. Kim-Spoon J. (2021). A systematic review of associations between adverse peer experiences and emotion regulation in adolescence. Clin. Child. Fam. Psychol. Rev. 24, 141–163. doi: 10.1007/s10567-020-00337-x, PMID: 33428070

[ref52] Houltberg B. J. Henry C. S. Morris A. S. (2012). Family interactions, exposure to violence, and emotion regulation: perceptions of children and early adolescents at risk. Fam. Relat. 61, 283–296. doi: 10.1111/j.1741-3729.2011.00699.x

[ref53] Hu L. Bentler P. M. (1999). Cutoff criteria for fit indexes in covariance structure analysis: conventional criteria versus new alternatives. Struct. Equ. Model. Multidiscip. J. 6, 1–55. doi: 10.1080/10705519909540118

[ref54] Hughes E. K. Gullone E. (2008). Internalizing symptoms and disorders in families of adolescents: a review of family systems literature. Clin. Psychol. Rev. 28, 92–117. doi: 10.1016/j.cpr.2007.04.002, PMID: 17509739

[ref001] IBM Corp. Released (2019). IBM SPSS Statistics for Windows, Version 26.0. Armonk, NY: IBM Corp., PMID:

[ref55] Jaffe M. Gullone E. Hughes E. K. (2010). The roles of temperamental dispositions and perceived parenting behaviours in the use of two emotion regulation strategies in late childhood. J. Appl. Dev. Psychol. 31, 47–59. doi: 10.1016/j.appdev.2009.07.008

[ref56] Johnson P. Waldo M. (1998). Integrating Minuchin’s boundary continuum and Bowen’s differentiation scale: a curvilinear representation. Contemp. Fam. Ther. 20, 403–413. doi: 10.1023/A:1022429332033

[ref57] Kealy D. Laverdière O. Cox D. W. Hewitt P. L. (2020). Childhood emotional neglect and depressive and anxiety symptoms among mental health outpatients: the mediating roles of narcissistic vulnerability and shame. J. Ment. Health 1–9. doi: 10.1080/09638237.2020.1836557 [Epub ahead of print]. 33084445

[ref58] Kim S. Thibodeau R. Jorgensen R. S. (2011). Shame, guilt, and depressive symptoms: a meta-analytic review. Psychol. Bull. 137, 68–96. doi: 10.1037/a0021466, PMID: 21219057

[ref59] Kivisto K. L. Welsh D. P. Darling N. Culpepper C. L. (2015). Family enmeshment, adolescent emotional dysregulation, and the moderating role of gender. J. Fam. Psychol. 29, 604–613. doi: 10.1037/fam0000118, PMID: 26374939

[ref60] Kronmüller K.-T. Von Maltzahn H. Horn H. Hartmann M. Victor D. Reck C.. (2008). Zur Erfassung von Schuld und Scham bei Kindern und Jugendlichen [On the assessment of guilt and shame in children and adolescents]. PPmP-Psychother. · Psychosom. · Med. Psychol 58, 313–320. doi: 10.1055/s-2007-986359 18642405

[ref61] Laursen B. Collins W. A. (2009). “Parent-child relationships during adolescence,” in Handbook of Adolescent Psychology: Contextual Influences on Adolescent Development. eds. Lerner R. M. Steinberg L. (Hoboken, NJ, United States: John Wiley & Sons, Inc.), 3–42.

[ref62] Leach C. W. (2017). “Understanding shame and guilt,” in Handbook of the psychology of Self-Forgiveness. eds. Woodyatt L. Worthington E. Griffin B. (Berlin: Springer), 17–28.

[ref63] Leach C. W. Cidam A. (2015). When is shame linked to constructive approach orientation? A meta-analysis. J. Pers. Soc. Psychol. 109, 983–1002. doi: 10.1037/pspa0000037, PMID: 26641074

[ref64] Lee J. (2020). Mental health effects of school closures during COVID-19. Lancet Child Adolesc. Health 4, 421. doi: 10.1016/S2352-4642(20)30109-7, PMID: 32302537 PMC7156240

[ref65] Leiner D. J. (2019). SoSci Survey. Available at: https://www.soscisurvey.de (Accessed July 7, 2022).

[ref66] Leung F. Schwartzman A. Steiger H. (1996). Testing a dual-process family model in understanding the development of eating pathology: a structural equation modeling analysis. Int. J. Eat. Disord. 20, 367–375. doi: 10.1002/(SICI)1098-108X(199612)20:4<367::AID-EAT4>3.0.CO;2-L, PMID: 8953324

[ref67] Lewis H. B. (1971). Shame and Guilt in Neurosis. New York, NY: International Univ. Press. 5150685

[ref68] Loader P. (1998). Such a shame—a consideration of shame and shaming mechanisms in families. Child Abuse Rev. 7, 44–57. doi: 10.1002/(SICI)1099-0852(199801/02)7:1<44::AID-CAR334>3.0.CO;2-7

[ref69] Lohbeck A. Schultheiß J. Petermann F. Petermann U. (2015). Die deutsche Selbstbeurteilungsversion des Strengths and Difficulties Questionnaire (SDQ-Deu-S): Psychometrische Eigenschaften, Faktorenstruktur und Grenzwerte [The german self-assessment version of the strengths and difficulties questionnaire (SDQ-Deu-S): psychometric properties, factor structure, and cutoff values]. Diagnostica 61, 222–235. doi: 10.1026/0012-1924/a000153

[ref70] Luyten P. Fontaine J. R. Corveleyn J. (2002). Does the Test of Self-Conscious Affect (TOSCA) measure maladaptive aspects of guilt and adaptive aspects of shame? An empirical investigation. Personal. Individ. Differ 33, 1373–1387. doi: 10.1016/S0191-8869(02)00197-6

[ref71] Manion J. C. (2002). The moral relevance of shame. Am. Philos. Q. 39, 73–90.

[ref72] Maxwell S. E. Cole D. A. Mitchell M. A. (2011). Bias in cross-sectional analyses of longitudinal mediation: partial and complete mediation under an autoregressive model. Multivar. Behav. Res. 46, 816–841. doi: 10.1080/00273171.2011.606716, PMID: 26736047

[ref73] Miller K. E. King C. A. Shain B. N. Naylor M. W. (1992). Suicidal adolescents’ perceptions of their family environment. Suicide Life Threat. Behav. 22, 226–239. 1626334

[ref74] Mills R. S. L. (2005). Taking stock of the developmental literature on shame. Dev. Rev. 25, 26–63. doi: 10.1016/j.dr.2004.08.001

[ref75] Morris A. S. Houltberg B. J. Criss M. M. Bosler C. D. (2017). “Family context and psychopathology: The mediating role of children’s emotion regulation,” in The Wiley Handbook of Developmental Psychopathology. eds. Centifanti L. C. Williams D. M. (Chichester, UK: John Wiley & Sons, Ltd), 365–389.

[ref76] Morris A. S. Silk J. S. Steinberg L. Myers S. S. Robinson L. R. (2007). The role of the family context in the development of emotion regulation. Soc. Dev. 16, 361–388. doi: 10.1111/j.1467-9507.2007.00389.x, PMID: 19756175 PMC2743505

[ref77] Muris P. Meesters C. (2014). Small or big in the eyes of the other: on the developmental psychopathology of self-conscious emotions as shame, guilt, and pride. Clin. Child. Fam. Psychol. Rev. 17, 19–40. doi: 10.1007/s10567-013-0137-z, PMID: 23712881

[ref78] Muris P. Meesters C. Cima M. Verhagen M. Brochard N. Sanders A.. (2014). Bound to feel bad about oneself: relations between attachment and the self-conscious emotions of guilt and shame in children and adolescents. J. Child Fam. Stud. 23, 1278–1288. doi: 10.1007/s10826-013-9817-z

[ref79] Murphy S. E. Boyd-Soisson E. Jacobvitz D. B. Hazen N. L. (2017). Dyadic and triadic family interactions as simultaneous predictors of children’s externalizing behaviors: family predictors of externalizing symptoms. Fam. Relat. 66, 346–359. doi: 10.1111/fare.12225

[ref80] Murray C. Waller G. Legg C. (2000). Family dysfunction and bulimic psychopathology: the mediating role of shame. Int. J. Eat. Disord. 28, 84–89. doi: 10.1002/(SICI)1098-108X(200007)28:1<84::AID-EAT10>3.0.CO;2-R, PMID: 10800017

[ref81] Muthén L. K. Muthén B. O. (2017). Mplus: Statistical Analysis With Latent Variables: User’s Guide *(Version 8)*. Los Angeles, CA: Authors.

[ref82] Nitkowski D. Fern J. Petermann U. Petermann F. Zeman J. L. (2019). Factorial structure of the german version of the emotion expression scale for children in early adolescents. J. Psychoeduc. Assess. 37, 517–523. doi: 10.1177/0734282918773478

[ref83] O’Leary J. L. McKee L. G. Faro A. L. (2019). Guilt and shame: explaining associations between emotion socialization and emerging adult well-being. Fam. Relat. 68, 608–623. doi: 10.1111/fare.12394

[ref84] Olson D. H. (1989). Family assessment and intervention: the circumplex model of family systems. Child Youth Serv. 11, 9–48. doi: 10.1300/J024v11n01_02, PMID: 30730296

[ref85] Olson D. H. (2000). Circumplex model of marital and family systems. J. Fam. Ther. 22, 144–167. doi: 10.1111/1467-6427.00144 6840263

[ref86] Olson D. H. (2011). FACES IV and the circumplex model: validation study. J. Marital. Fam. Ther. 37, 64–80. doi: 10.1111/j.1752-0606.2009.00175.x, PMID: 21198689

[ref87] Parise M. Canzi E. Olivari M. G. Ferrari L. (2019). Self-concept clarity and psychological adjustment in adolescence: the mediating role of emotion regulation. Personal. Individ. Differ. 138, 363–365. doi: 10.1016/j.paid.2018.10.023

[ref88] Penza-Clyve S. Zeman J. (2002). Initial validation of the emotion expression scale for children (EESC). J. Clin. Child Adolesc. Psychol. 31, 540–547. doi: 10.1207/S15374424JCCP3104_12, PMID: 12402572

[ref89] Pinquart M. (2017). Associations of parenting dimensions and styles with externalizing problems of children and adolescents: an updated meta-analysis. Dev. Psychol. 53, 873–932. doi: 10.1037/dev0000295, PMID: 28459276

[ref90] Pulakos J. (1996). Family environment and shame: is there a relationship? J. Clin. Psychol. 52, 617–623. doi: 10.1002/(SICI)1097-4679(199611)52:6<617::AID-JCLP3>3.0.CO;2-H, PMID: 8912104

[ref91] Rabinowitz J. A. Osigwe I. Drabick D. A. G. Reynolds M. D. (2016). Negative emotional reactivity moderates the relations between family cohesion and internalizing and externalizing symptoms in adolescence. J. Adolesc. 53, 116–126. doi: 10.1016/j.adolescence.2016.09.007, PMID: 27718379 PMC5116243

[ref92] Ravens-Sieberer U. Kaman A. Erhart M. Devine J. Schlack R. Otto C. (2021). Impact of the COVID-19 pandemic on quality of life and mental health in children and adolescents in Germany. Eur. Child Adolesc. Psychiatry 31, 879–889. doi: 10.1007/s00787-021-01889-1, PMID: 33492480 PMC7829493

[ref93] Repetti R. L. Taylor S. E. Seeman T. E. (2002). Risky families: family social environments and the mental and physical health of offspring. Psychol. Bull. 128, 330–366. doi: 10.1037/0033-2909.128.2.330, PMID: 11931522

[ref94] Riediger M. Klipker K. (2014). “Emotion regulation in adolescence,” in Handbook of Emotion Regulation. ed. Gross J. J. (New York, NY, United States: Guilford Publications), 187–202.

[ref95] Rollins E. M. Crandall A. (2021). Self-regulation and shame as mediators between childhood experiences and young adult health. Front. Psych. 12:649911. doi: 10.3389/fpsyt.2021.649911, PMID: 33935835 PMC8085257

[ref96] Rothstein A. M. (1994). Shame and the superego: clinical and theoretical considerations. Psychoanal. Study Child 49, 263–277. doi: 10.1080/00797308.1994.11823063 7809288

[ref97] Rowsell M. Doyle S. Francis S. E. (2016). The role of BIS sensitivity in the relationship between family enmeshment and child anxiety. J. Child Fam. Stud. 25, 2585–2596. doi: 10.1007/s10826-016-0415-8

[ref98] Schibalski J. V. Müller M. Ajdacic-Gross V. Vetter S. Rodgers S. Oexle N.. (2017). Stigma-related stress, shame and avoidant coping reactions among members of the general population with elevated symptom levels. Compr. Psychiatry 74, 224–230. doi: 10.1016/j.comppsych.2017.02.001, PMID: 28236772

[ref99] Schoenleber M. Berenbaum H. (2012). Shame regulation in personality pathology. J. Abnorm. Psychol. 121, 433–446. doi: 10.1037/a0025281, PMID: 21895346

[ref100] Shaffer A. Suveg C. Thomassin K. Bradbury L. L. (2012). Emotion socialization in the context of family risks: links to child emotion regulation. J. Child Fam. Stud. 21, 917–924. doi: 10.1007/s10826-011-9551-3, PMID: 28493749

[ref101] Shiffman S. Stone A. A. Hufford M. R. (2008). Ecological momentary assessment. Annu. Rev. Clin. Psychol. 4, 1–32. doi: 10.1146/annurev.clinpsy.3.022806.091415, PMID: 18509902

[ref102] Simpson E. G. Vannucci A. Ohannessian C. M. (2018). Family functioning and adolescent internalizing symptoms: a latent profile analysis. J. Adolesc. 64, 136–145. doi: 10.1016/j.adolescence.2018.02.004, PMID: 29471247

[ref103] Smetana J. G. Robinson J. Rote W. M. (2014). “Socialization in adolescence,” in Handbook of Socialization, Second Edition: Theory and Research. eds. Grusec J. E. Hastings P. D. (New York: Guilford Publications), 60–84.

[ref104] Smiley P. A. Rasmussen H. F. Buttitta K. V. Hecht H. K. Scharlach K. M. Borelli J. L. (2020). Parent control and child shame: associations with children’s task persistence and depressive symptoms in middle childhood. Parenting 20, 311–336. doi: 10.1080/15295192.2019.1694837

[ref105] Soloski K. L. Berryhill M. B. (2016). Gender differences: emotional distress as an indirect effect between family cohesion and adolescent alcohol use. J. Child Fam. Stud. 25, 1269–1283. doi: 10.1007/s10826-015-0311-7

[ref106] Southam-Gerow M. A. Kendall P. C. (2002). Emotion regulation and understanding: implications for child psychopathology and therapy. Clin. Psychol. Rev. 22, 189–222. doi: 10.1016/S0272-7358(01)00087-3, PMID: 11806019

[ref107] Spear L. P. (2009). Heightened stress responsivity and emotional reactivity during pubertal maturation: implications for psychopathology. Dev. Psychopathol. 21, 87–97. doi: 10.1017/S0954579409000066, PMID: 19144224 PMC3036838

[ref108] Stappenbeck J. Wendell A. Bröning S. Stolle M. (2006). FACES IV Testmappe: Deutsche Bearbeitung [FACES IV Test Folder: German Adaptation]. Minneapolis: Life Innovations.

[ref109] Stark K. D. Humphrey L. L. Crook K. Lewis K. (1990). Perceived family environments of depressed and anxious children: child’s and maternal figure’s perspectives. J. Abnorm. Child Psychol. 18, 527–547. doi: 10.1007/BF00911106, PMID: 2266224

[ref110] Steeger C. M. Gondoli D. M. (2013). Mother–adolescent conflict as a mediator between adolescent problem behaviors and maternal psychological control. Dev. Psychol. 49, 804–814. doi: 10.1037/a0028599, PMID: 22612432 PMC4203150

[ref111] Steffen A. Akmatov M. K. Holstiege J. Bätzing J. (2018). Diagnoseprävalenz psychischer Störungen bei Kindern und Jugendlichen in Deutschland: eine Analyse bundesweiter vertragsärztlicher Abrechnungsdaten der Jahre 2009 bis 2017 [Diagnostic prevalence of mental disorders in children and adolescents in Germany: An analysis of nationwide billing data from panel physicians from 2009 to 2017]. Zentralinstitut für die kassenärztliche Versorgung in Deutschland (Zi). doi: 10.20364/VA-18.07

[ref112] Stuewig J. McCloskey L. A. (2005). The relation of child maltreatment to shame and guilt among adolescents: psychological routes to depression and delinquency. Child Maltreat. 10, 324–336. doi: 10.1177/1077559505279308, PMID: 16204735

[ref113] Szentágotai-Tătar A. Miu A. C. (2016). Individual differences in emotion regulation, childhood trauma and proneness to shame and guilt in adolescence. PLoS One 11:e0167299. doi: 10.1371/journal.pone.0167299, PMID: 27898709 PMC5127568

[ref114] Tafà M. Cimino S. Ballarotto G. Bracaglia F. Bottone C. Cerniglia L. (2017). Female adolescents with eating disorders, parental psychopathological risk and family functioning. J. Child Fam. Stud. 26, 28–39. doi: 10.1007/s10826-016-0531-5, PMID: 29042824

[ref115] Talmon A. Ginzburg K. (2017). Between childhood maltreatment and shame: the roles of self-objectification and disrupted body boundaries. Psychol. Women Q. 41, 325–337. doi: 10.1177/0361684317702503

[ref116] Tangney J. P. Stuewig J. Mashek D. J. (2007). Moral emotions and moral behavior. Annu. Rev. Psychol. 58, 345–372. doi: 10.1146/annurev.psych.56.091103.070145, PMID: 16953797 PMC3083636

[ref117] Tangney J. P. Wagner P. E. Gramzow R. (1991). The Test of Self-Conscious Affect for Adolescents (TOSCA-A). Fairfax, VA: George Mason University.

[ref118] Tangney J. P. Wagner P. Gramzow R. (1992). Proneness to shame, proneness to guilt, and psychopathology. J. Abnorm. Psychol. 101, 469. doi: 10.1037/0021-843X.101.3.469, PMID: 1500604

[ref119] Teachman B. A. Allen J. P. (2007). Development of social anxiety: social interaction predictors of implicit and explicit fear of negative evaluation. J. Abnorm. Child Psychol. 35, 63–78. doi: 10.1007/s10802-006-9084-1, PMID: 17171538 PMC3395171

[ref120] Thompson R. A. (2014). “Socialization of emotion and emotion regulation in the family,” in Handbook of Emotion Regulation. ed. Gross J. J. (New York, NY, United States: Guilford Publications), 173–186.

[ref121] Tracy J. L. Robins R. W. (2004). Putting the self into self-conscious emotions: a theoretical model. Psychol. Inq. 15, 103–125. doi: 10.1207/s15327965pli1502_01

[ref122] Trosper S. E. Buzzella B. A. Bennett S. M. Ehrenreich J. T. (2009). Emotion regulation in youth with emotional disorders: implications for a unified treatment approach. Clin. Child. Fam. Psychol. Rev. 12, 234–254. doi: 10.1007/s10567-009-0043-6, PMID: 19238542

[ref123] van Dijk R. van der Valk I. E. Deković M. Branje S. (2020). A meta-analysis on interparental conflict, parenting, and child adjustment in divorced families: Examining mediation using meta-analytic structural equation models. Clin. Psychol. Rev. 79:101861. doi: 10.1016/j.cpr.2020.101861, PMID: 32512420

[ref124] van Eickels R. L. Zemp M. Grütter J. (2022). Familiäre Unterstützung als Schutzfaktor für Jugendliche während der COVID-19-Pandemie [Family support as a protective factor for adolescents during the COVID-19 pandemic]. Kindh. Entwickl. 31, 111–118. doi: 10.1026/0942-5403/a000376

[ref125] Velotti P. Elison J. Garofalo C. (2014). Shame and aggression: different trajectories and implications. Aggress. Violent Behav. 19, 454–461. doi: 10.1016/j.avb.2014.04.011

[ref126] Velotti P. Garofalo C. Bottazzi F. Caretti V. (2017). Faces of shame: implications for self-esteem, emotion regulation, aggression, and well-being. Aust. J. Psychol. 151, 171–184. doi: 10.1080/00223980.2016.1248809 27858531

[ref127] Walter J. L. Burnaford S. M. (2006). Developmental changes in adolescents’ guilt and shame: the role of family climate and gender. North Am. J. Psychol. 8, 321–338.

[ref128] Wang Y. Tian L. Guo L. Huebner E. S. (2020). Family dysfunction and adolescents’ anxiety and depression: a multiple mediation model. J. Appl. Dev. Psychol. 66:101090. doi: 10.1016/j.appdev.2019.101090

[ref129] Wark M. J. Kruczek T. Boley A. (2003). Emotional neglect and family structure: impact on student functioning. Child Abuse Negl. 27, 1033–1043. doi: 10.1016/S0145-2134(03)00162-5, PMID: 14550330

[ref130] Watson W. H. (2012). “Family systems,” in Encyclopedia of Human Behavior. 2nd Edn. Ramachandran V. S. (San Diego: Academic Press), 184–193.

[ref131] Wojcik K. D. Cox D. W. Kealy D. (2019). Adverse childhood experiences and shame-and guilt-proneness: examining the mediating roles of interpersonal problems in a community sample. Child Abuse Negl. 98:104233. doi: 10.1016/j.chiabu.2019.104233, PMID: 31669776

[ref132] Wong Y. Tsai J. (2007). “Cultural models of shame and guilt,” in The Self-Conscious Emotions: Theory and Research. eds. Tracy J. L. Robins R. W. Tangney J. P. (New York: Guilford Press), 209–223.

[ref133] Young R. Lennie S. Minnis H. (2011). Children’s perceptions of parental emotional neglect and control and psychopathology: children’s perceptions of parental neglect and control. J. Child Psychol. Psychiatry 52, 889–897. doi: 10.1111/j.1469-7610.2011.02390.x, PMID: 21438874 PMC3170712

[ref134] Zahn-Waxler C. Shirtcliff E. A. Marceau K. (2008). Disorders of childhood and adolescence: gender and psychopathology. Annu. Rev. Clin. Psychol. 4, 275–303. doi: 10.1146/annurev.clinpsy.3.022806.091358, PMID: 18370618

[ref135] Zimmermann P. Iwanski A. (2014). Emotion regulation from early adolescence to emerging adulthood and middle adulthood: age differences, gender differences, and emotion-specific developmental variations. Int. J. Behav. Dev. 38, 182–194. doi: 10.1177/0165025413515405

