# Supplementary material for: Family cohesion, shame-proneness, expressive suppression, and adolescent mental health—A path model approach
Source: Front Psychol. 2022 Aug 3;13:921250. doi: 10.3389/fpsyg.2022.921250 (PMC9382198; doi:10.3389/fpsyg.2022.921250)
Supplement: Supplementary file 1 [file Data_Sheet_1.PDF]

Supplementary Material

1 Supplementary Tables

1.1 Intercorrelations

Table S1

Intercorrelations, Means, and Standard Deviations of the Final Sample including Maladaptive Shame-Proneness

Variable	COH	SP	MALSP	ES	INT	EXT	GP	Age
COH	2.10 (1.16)							
SP	**-.230****	3.21 (0.75)						
MALSP	**-.253****	**.990****	3.10 (0.79)					
ES	**-.377****	**.555****	**.570****	3.18 (0.87)				
INT	**-.363****	**.632****	**.648****	**.642****	0.94 (0.42)			
EXT	**-.347****	**.214****	**.240****	**.341****	**.317****	0.63 (0.34)		
GP	**.144****	**.458****	**.416****	**.162****	**.214****	-.052	3.94 (0.51)	
Age	-.009	-.058	-.058	.001	.015	-.070	.081	15.74 (1.22)

Note. COH = family cohesion, SP = shame-proneness, MALSP = maladaptive shame-proneness (excluding items 7 and 12 of the TOSCA-A), ES = expressive suppression, INT = internalizing problems, EXT = externalizing problems, GP = guilt-proneness. Means and standard deviations of the respective variables are displayed on the table diagonal (*M* (*SD*)). Significant values are in bold. $^{**}p < .001$.

1.2 Control Path Model with Maladaptive Shame-Proneness

Table S2

Direct Effects of the Path Model with Maladaptive Shame-Proneness

Direct Effects	*b*	*(SE)*	95% CI	*p*	β	*(SE)*	95% CI	*p*	R^2
Internalizing Problems									.563
COH	**-.048**	(.011)	[-.069; -.026]	< .001	**-.132**	(.031)	[-.193; -.071]	< .001	
MALSP	**.198**	(.020)	[.160; .237]	< .001	**.377**	(.037)	[.303; .450]	< .001	
ES	**.167**	(.018)	[.131; .203]	< .001	**.350**	(.038)	[.275; .424]	< .001	
Age	.008	(.010)	[-.012; .028]	.439	.023	(.030)	[-.036; .083]	.439	
Gender (F vs. M)	**-.144**	(.029)	[-.201; -.086]	< .001	**-.141**	(.029)	[-.197; -.085]	< .001	
Gender (F vs. O)	-.004	(.061)	[-.124; .117]	.952	.001	(.022)	[-.044; .042]	.952	
Externalizing Problems									.187
COH	**-.073**	(.012)	[-.097; -.049]	< .001	**-.254**	(.042)	[-.336; -.172]	< .001	
MALSP	.019	(.021)	[-.022; .059]	.372	.044	(.049)	[-.052; .140]	.373	
ES	**.083**	(.020)	[.044; .122]	< .001	**.215**	(.051)	[.115; .316]	< .001	
Age	-.016	(.011)	[-.038; .006]	.152	-.059	(.041)	[-.139; .021]	.151	
Gender (F vs. M)	.003	(.033)	[-.061; .068]	.922	.004	(.040)	[-.075; .083]	.922	
Gender (F vs. O)	**.214**	(.083)	[.053; .376]	.009	**.095**	(.039)	[.019; .172]	.014	
Expressive Suppression									.387
COH	**-.189**	(.027)	[-.242; -.135]	< .001	**-.251**	(.036)	[-.321; -.180]	< .001	

MALSP	**.546**	(.040)	[.467; .624]	< .001	**.494**	(.034)	[.427; .562]	< .001	
Age	.025	(.025)	[-.024; .074]	.319	.035	(.037)	[-.034; .103]	.320	
Gender (F vs. M)	-.090	(.080)	[-.247; .068]	.264	-.042	(.035)	[-.116; .031]	.262	
Gender (F vs. O)	.132	(.207)	[-.211; .599]	.348	.033	(.039)	[-.037; .103]	.352	
Maladaptive Shame-Proneness									.330
COH	**-.210**	(.028)	[-.264; -.156]	< .001	**-.308**	(.040)	[-.387; -.229]	< .001	
GP	**.667**	(.055)	[.560; .774]	< .001	**.430**	(.034)	[.363; .497]	< .001	
Age	**-.084**	(.024)	[-.131; -.038]	< .001	**-.131**	(.037)	[-.202; -.059]	< .001	
Gender (F vs. M)	**-.419**	(.066)	[-.549; -.289]	< .001	**-.217**	(.035)	[-.284; -.149]	< .001	
Gender (F vs. O)	.132	(.208)	[-.275; .538]	.526	.025	(.039)	[-.052; .102]	.527	

Note. b (SE) = unstandardized predictor and standard error, β *(SE)* = standardized predictor and standard error, COH = family cohesion, MALSP = maladaptive shame-proneness (excluding items 7 and 12 of the TOSCA-A), GP = guilt-proneness, ES = expressive suppression. Gender was dummy-coded with the reference group "female": F vs. M = female vs. male, F vs. O = female vs. other gender. Significant values are in bold. Model fit: CFI = .999, TLI = .994, SRMR = .013, RMSEA = .021, 90% CI [.000; .079], *p* =.725.

Table S3

Indirect Effects of the Path Model with Maladaptive Shame-Proneness

Total and Indirect Effects	*b* (*SE*)	95% CI	β (*SE*)	95% CI	*p*
Internalizing Problems					
Total Effect	**-.140** (.014)	[-.166; -.113]	**-.389** (.037)	[-.461; -.317]	< .001
Total Indirect Effect	**-.092** (.011)	[-.113; -.071]	**-.257** (.029)	[-.314; -.200]	< .001
1. COH → MALSP → INT	**-.042** (.007)	[-.055; -.028]	**-.116** (.018)	[-.152; -.080]	< .001
2. COH → ES → INT	**-.031** (.006)	[-.043; -.020]	**-.088** (.016)	[-.119; -.057]	< .001
3. COH → MALSP → ES → INT	**-.019** (.004)	[-.026; -.012]	**-.053** (.010)	[-.072; -.034]	< .001
Externalizing Problems					
Total Effect	**-.102** (.012)	[-.125; -.080]	**-.354** (.038)	[-.429; -.279]	< .001
Total Indirect Effect	**-.029** (.006)	[-.040; -.018]	**-.100** (.020)	[-.139; -.062]	< .001
4. COH → MALSP→ EXT	-.004 (.004)	[-.012; .005]	-.013 (.015)	[-.043; .016]	.370
5. COH → ES → EXT	**-.016** (.004)	[-.024; -.007]	**-.054** (.015)	[-.083; -.025]	< .001
6. COH → MALSP→ ES → EXT	**-.009** (.003)	[-.014; -.004]	**-.033** (.009)	[-.050; -.016]	< .001

Note. Model fit: CFI = .999, TLI = .994, SRMR = .013, RMSEA = .021, 90% CI [.000; .079], *p* =.725. *b (SE)* = unstandardized predictor and standard error, β *(SE)* = standardized predictor and standard error, COH = family cohesion, MALSP = maladaptive shame-proneness (excluding items 7 and 12 of the TOSCA-A), ES = expressive suppression, INT = internalizing problems, EXT = externalizing problems. Significant values are in bold. Model fit: CFI = .999, TLI = .994, SRMR = .013, RMSEA = .021, 90% CI [.000; .079], *p* =.725.

1.3 Control Path Model with Guilt-Proneness

Table S4

Direct Effects of the Path Model with Guilt-Proneness

Direct Effects	*b*	*(SE)*	95% CI	*p*	β	*(SE)*	95% CI	*p*	R^2
Internalizing Problems									.559
COH	**-.048**	(.012)	[-.071; -.026]	< .001	**-.135**	(.032)	[-.198; -.071]	< .001	
GP	-.020	(.028)	[-.075; .034]	.465	-.025	(.034)	[-.092; .042]	.465	
ES	**.171**	(.018)	[.135; .207]	< .001	**.358**	(.038)	[.284; .432]	< .001	
SP	**.207**	(.023)	[.162; .252]	< .001	**.374**	(.041)	[.294; .453]	< .001	
Age	.007	(.010)	[-.014; .027]	.521	.020	(.030)	[-.040; .079]	.521	
Gender (F vs. M)	**-.152**	(.029)	[-.210; -.095]	< .001	**-.149**	(.029)	[-.206; -.093]	< .001	
Gender (F vs. O)	.011	(.061)	[-.109; .131]	.854	.004	(.022)	[-.039; .047]	.855	
Externalizing Problems									.191
COH	**-.068**	(.013)	[-.093; -.043]	< .001	**-.235**	(.044)	[-.321; -.149]	< .001	
GP	-.055	(.034)	[-.122; .013]	.111	-.083	(.052)	[-.186; .020]	.113	
ES	**.086**	(.020)	[.047; .125]	< .001	**.223**	(.051)	[.123; .323]	< .001	
SP	.029	(.025)	[-.020; .078]	.249	.065	(.057)	[-.046; .176]	.250	
Age	-.014	(.011)	[-.037; .008]	.203	-.052	(.041)	[-.133; .028]	.201	
Gender (F vs. M)	-.003	(.033)	[-.067; .061]	.923	-.004	(.040)	[-.082; .075]	.923	
Gender (F vs. O)	**.217**	(.083)	[.055; .380]	.009	**.097**	(.039)	[.020; .174]	.014	

Expressive Suppression									.380
COH	**-.191**	(.029)	[-.248; -.134]	< .001	**-.253**	(.039)	[-.329; -.178]	< .001	
GP	-.078	(.075)	[-.225; .069]	.300	-.045	(.044)	[-.131; .040]	.298	
SP	**.582**	(.051)	[.483; .682]	< .001	**.502**	(.042)	[.420; .583]	< .001	
Age	.022	(.026)	[-.028; .073]	.384	.031	(.036)	[-.039; .102]	.384	
Gender (F vs. M)	-.116	(.080)	[-.273; .042]	.150	-.054	(.038)	[-.128; .019]	.149	
Gender (F vs. O)	.238	(.211)	[-.175; .652]	.259	.041	(.037)	[-.031; .112]	.264	
Guilt-Proneness									.288
COH	**.115**	(.017)	[.082; .148]	< .001	**.262**	(.037)	[.189; .334]	< .001	
SP	**.349**	(.029)	[.293; .405]	< .001	**.514**	(.036)	[.445; .584]	< .001	
Age	**.043**	(.016)	[.011; .076]	.008	**.104**	(.039)	[.028; .180]	.007	
Gender (F vs. M)	-.041	(.050)	[-.138; .057]	.413	-.033	(.040)	[-.110; .045]	.413	
Gender (F vs. O)	.029	(.080)	[-.128; .187]	.715	.009	(.024)	[-.038; .055]	.715	

Note. b (SE) = unstandardized predictor and standard error, β *(SE)* = standardized predictor and standard error, COH = family cohesion, GP = guilt-proneness, SP = shame-proneness, ES = expressive suppression. Gender was dummy-coded with the reference group "female": F vs. M = female vs. male, F vs. O = female vs. other gender. Significant values are in bold. No model fit indices provided due to model saturation.

Table S5

Indirect Effects of the Path Model with Guilt-Proneness

Total and Indirect Effects	*b* (*SE*)	95% CI	β (*SE*)	95% CI	*p*
Internalizing Problems					
Total Effect	**-.085** (.012)	[-.108; -.062]	**-.236** (.032)	[-.299; -.173]	< .001
Total Indirect Effect	**-.036** (.006)	[-.050; -.023]	**-.102** (.019)	[-.139; -.064]	< .001
1. COH → GP → INT	-.002 (.004)	[-.009; .004]	-.007 (.009)	[-.024; -.011]	.470
2. COH → ES → INT	**-.033** (.004)	[-.045; -.021]	**-.091** (.017)	[-.124; -.057]	< .001
3. COH → GP → ES → INT	-.002 (.001)	[-.004; .001]	-.004 (.004)	[-.012; .004]	.303
Externalizing Problems					
Total Effect	**-.091** (.012)	[-.115; -.068]	**-.316** (.041)	[-.396; -.236]	< .001
Total Indirect Effect	**-.023** (.006)	[-.035; -.012]	**-.081** (.020)	[-.120; -.042]	< .001
4. COH → GP→ EXT	-.006 (.004)	[-.014; .002]	-.022 (.014)	[-.049; .006]	.125
5. COH → ES → EXT	**-.016** (.004)	[-.025; -.008]	**-.057** (.015)	[-.087;-.027]	< .001
6. COH → GP→ ES → EXT	-.001 (.001)	[-.002; .001]	-.003 (.003)	[-.008; .003]	.315

Note. b (SE) = unstandardized predictor and standard error, β *(SE)* = standardized predictor and standard error, COH = family cohesion, GP = guilt-proneness, ES = expressive suppression, INT = internalizing problems, EXT = externalizing problems. Significant values are in bold. No model fit indices provided due to model saturation.
